# Expression of Human Gaucher Disease Gene GBA Generates Neurodevelopmental Defects and ER Stress in *Drosophila* Eye

**DOI:** 10.1371/journal.pone.0069147

**Published:** 2013-08-02

**Authors:** Takahiro Suzuki, Masami Shimoda, Kumpei Ito, Shuji Hanai, Hidenobu Aizawa, Tomoki Kato, Kazunori Kawasaki, Terumi Yamaguchi, Hyung Don Ryoo, Naoko Goto-Inoue, Mitsutoshi Setou, Shoji Tsuji, Norio Ishida

**Affiliations:** 1 National Institute of Advanced Industrial Science and Technology, Tsukuba, Ibaraki, Japan; 2 Graduate School of Life and Environmental Sciences, University of Tsukuba, Ibaraki, Japan; 3 National Institute of Agrobiological Sciences, Tsukuba, Ibaraki, Japan; 4 Department of Cell Biology, New York University School of Medicine, New York, New York, United States of America; 5 Graduate School of Health Promotion Sciences, Tokyo Metropolitan University, Tokyo, Japan; 6 Department of Cell Biology and Anatomy, Hamamatsu University School of Medicine, Shizuoka, Japan; 7 Department of Neurology, The University of Tokyo, Graduate School of Medicine, Tokyo, Japan; University Hospital S. Maria della Misericordia, Udine, Italy

## Abstract

Gaucher disease (GD) is the most common of the lysosomal storage disorders and is caused by defects in the GBA gene encoding glucocerebrosidase (GlcCerase). The accumulation of its substrate, glucocylceramide (GlcCer) is considered the main cause of GD. We found here that the expression of human mutated GlcCerase gene (hGBA) that is associated with neuronopathy in GD patients causes neurodevelopmental defects in *Drosophila* eyes. The data indicate that endoplasmic reticulum (ER) stress was elevated in *Drosophila* eye carrying mutated hGBAs by using of the ER stress markers dXBP1 and dBiP. We also found that Ambroxol, a potential pharmacological chaperone for mutated hGBAs, can alleviate the neuronopathic phenotype through reducing ER stress. We demonstrate a novel mechanism of neurodevelopmental defects mediated by ER stress through expression of mutants of human GBA gene in the eye of *Drosophila*.

## Introduction

Individuals with Gaucher disease (GD) are deficient in the membrane-associated lysosomal enzyme, glucocerebrosidase (GlcCerase). This reticuloendothelial storage disorder is clinically classified as types 1 (chronic, nonneuronopathic), 2 (acute, neuronopathic) and 3 (chronic, neuronopathic) [1]. Almost 300 mutations have been identified in the human GlcCerase gene (hGBA) [2]. The R120W mutation results in mild disease [3], whereas the L444P mutation is associated with neurological abnormalities [4]–[9] and the complex allele RecNciI (L444P + A456P + V460V) is involved in acute neurological abnormalities [7], [9].

The general treatment of GD is to reduce the accumulation of stored glucocylceramide (GlcCer) substrate either by enhancing substrate degradation or by reducing its production. The main treatment strategy is intravenous enzyme replacement, which might partly restore a deficient enzymatic capacity [10]. However this strategy cannot prevent or treat neurological abnormalities, perhaps because GlcCerase cannot cross the blood–brain barrier [11] and therefore no strategies are currently available to treat the neurological abnormalities associated with GD.

Mouse models of GD were generated [12] by creating a GBA null allele [13], a point mutated GBA allele [14] or a GBA conditional knockout [15]. These models based the study on the notion that GD phenotypes are caused by accumulated stored GlcCer. Therefore, mutations or deletions were constructed from the endogenous homologous genes of mouse genome. In some cases, GlcCerase variants are retained to various degrees in the endoplasmic reticulum (ER) as seen in cells of patients with GD [16]. These findings indicated that mutated GlcCerase itself is toxic, but this is yet to be confirmed at molecular level.

*Drosophila* provides a flexible and powerful model with which to study neurodegenerative diseases [17]–[21] because most of the genetic pathways involved in normal development and diseases are conserved between *Drosophila* and mammals. Thus, understanding the molecular mechanisms of neurodegeneration in *Drosophila* might help to clarify human neurodegenerative processes [22]. Although several models for various neurodegenerative diseases such as Parkinson's disease have been created [23], a *Drosophila* model of GD is not available.

Here, we express mutated hGBA in the *Drosophila* eye using GMR-Gal4. We show that mutated hGBAs in particular, the RecNciI mutation that is associated with acute neurological abnormalities in humans, have neurodevelopmental defects in *Drosophila.* We also show that ER stress, which might contribute to neurodegeneration in many disorders [24], was increased in *Drosophila*. Furthermore, the expectorant Ambroxol was identified as a pharmacological chaperone for mutant hGBA [25] that could decrease ER stress and recover the morphological defects in *Drosophila*.

Our data suggest that the expression of mutant hGBA gene results in ER mediated ER stress and neurodevelopmental defects in *Drosophila* eye. Our *Drosophila* transgenic lines can serve as a powerful tool for investigating the mechanisms of neurodegeneration as well as novel therapeutic targets of GD.

## Materials and Methods

### Human GBA cDNAs

Human GBA cDNAs (WT, R120W and RecNciI) were generous gifts from Professor Shoji Tsuji at the University of Tokyo.

### Production of transgenic flies

Transgenic flies were generated as described [26] using pUAST vectors harboring hGBA cDNAs. The vectors were injected into yw *Drosophila melanogaster* embryos using the helper plasmid pπ25.7wc that encodes a transposase. One hGBA^WT^, two independent hGBA^R120W^ and three independent hGBA^RecNciI^ lines were generated. All recombinant DNA experiments proceeded under the approval of the AIST Recombinant DNA Committee.

### Isolation of RNA and quantitative RT-PCR

Flies were entrained at 25°C under LD (light:dark, 12:12 h) and then three-day-old male heads (Genotype: w;GMR-GAL4/CyO;UAS-hGBA) were analyzed. Male flies were normally entrained at 25°C under LD and continuously heat-shocked at 37°C twice daily for 0.5 h (at 9 am and 9 pm) for studies using the hs-GAL4 driver. Whole males (Genotype: w;hs-GAL4/CyO;UAS-hGBA/+) were collected three hours after the last shock. Fly heads or whole flies were homogenized in TRIzol reagent (Invitrogen, Carlsbad, California), mixed with 25% chloroform and then separated by centrifugation at 12,000×g for 15 min in 4°C. Supernatants were mixed with an equal volume of 2-propanol, separated by centrifugation at 12,000 g for 10 min at 4°C and then the pellets were mixed with 70% ethanol and separated by centrifugation at 7500×g for 5 min at 4°C. The pellets were mixed with dH_2_O. Complementary DNAs were synthesized using the Prime Script RT Reagent Kit (Takara Bio, Otsu, Japan) according to the manufacturer's protocol. The cDNA levels of the hGBA, dBiP and dRpL32 genes were measured by quantitative RT-PCR using a LightCycler (Roche Applied Science) with SYBR Premix Ex Taq (Takara Bio, Otsu, Japan). The amount of mRNA was corrected relative to that of dRpL32. Table 1 shows the sequences of the primer pairs.

**Table 1:** Primer sequences for Quantitative RT-PCR.

Gene	Forward and reverse sequences
hGBA	5′- TGG GCA GTG ACA GCT GAA -3′
hGBA	5′- CTG GAA GGG GTA TCC ACT CA -3′
dBiP	5′- GCT GGT GTT ATT GCC GGT CTG C -3′
dBiP	5′- GAT GCC TCG GGA TGG TTC CTT GC -3′
dRpL32	5′- AGA TCG TGA AGA AGC GCA CCA AG -3′
dRpL32	5′- CAC CAG GAA CTT CTT GAA TCC GG -3′

Human GBA primers were designed at Universal Probe Library Assay Design Center (Roche Applied Science).

Primers for dBiP [32] and dRpL32 [35] were as described in respective citations.

### Western blotting

Western blotting proceeded as described [26]. All transgenic combinations were entrained at 25°C under LD, and then the heads of flies with the w;GMR-GAL4/CyO;UAS-hGBA genotype collected at 11.00 a.m. were homogenized in extraction buffer containing 20 mM HEPES (pH 7.5), 100 mM KCl, 5% glycerol, 100 mM Na3VO4, 0.5 M EDTA, 0.1% Triton-X, 10 mg/mL antipain, 10 mg/mL pepstatin-A, 10 mg/mL leupeptin, 24 TIU/mL aprotinin and 0.1 M phenylmethyl-sulfonyl-fluoride (PMSF). The samples were separated by centrifugation at 20000×g for 5 min at 4°C. The protein concentration in each supernatant was determined using the BCA protein assay reagent (PIERCE, Rockville, MD, USA). The extracts were mixed with same volume of SDS-PAGE sample buffer containing 5% mercaptoethanol, boiled for three minutes and quickly cooled. Proteins (30 μg) from extracts resolved by electrophoresis on 10% SDS-PAGE gels were electrotransferred to ECL Hybond membranes (Amersham) using a carbon electrode for 90 min at 1 mA/cm^2^ and then probed for hGBA using the b55080 anti-GBA (1:2000) antibody (Abcam). Secondary HRP-labeled anti-mouse antibody was diluted 1:10,000 and signals were detected using ECL+^TM^ (Amersham).

### Scanning electron microscopy

Three-day-old males with the w;GMR-GAL4/CyO;UAS-hGBA genotype from each experimental transgenic were fixed in 2% glutaraldehyde/0.1 M phosphate buffered saline (PBS) for 12 h at 4°C. The samples were washed with 0.1 M PBS, sequentially dehydrated in 50%–100% ethanol and freeze-dried using t-butyl alcohol (VFD-20; Vacuum Device Inc., Mito, Japan). Dried samples were placed on a specimen stage and coated with osmium tetroxide using a PMC-5000 plasma ion coater (Meiwafosis Co., Tokyo, Japan). The *Drosophila* heads were examined by scanning electron microscopy (S-5000, Hitachi High-Technologies Co., Tokyo, Japan) at 5 kV.

Scanning electron microscopy proceeded as described [27] at 5 kV using a JSM-6301F (JEOL Ltd., Tokyo, Japan) scanning electron microscope. Three-day-old males with the w;GMR-GAL4/CyO;UAS-hGBA genotype from each experimental transgenic combinations were mounted on a stage with double-sided tape and sputter-coated with gold.

### Immunohistochemistry

All transgenic combinations were entrained at 25°C under LD, and then the eye imaginal discs of third instar larvae with the w;GMR-GAL4/UAS-xbp1-EGFP;UAS-hGBA/ TM6B genotype were fixed in Mildform 10N (Wako Pure Chemical Industries, Osaka, Japan) for 12 h at 4°C. The fixed discs were washed with PBST and probed for EGFP using the A6455 anti-GFP (1:2000) antibody (Invitrogen). Alexa Fluor 488 anti-rabbit secondary antibody was added and then the discs were examined by confocal laser scanning microscopy (Zeiss LSM700, Zeiss LSM5, OLYMPUS FV1000MPE). Values for fixed quantities of fluorescence intensity were measured using ImageJ.

### Ambroxol treatment

All transgenic combinations were maintained on yeast-glucose-agar medium containing Ambroxol hydrochloride (WAKO 013-18943) /DMSO (WAKO 043-07216) to final concentrations of 0 and 1 mM. The final concentration of DMSO in the medium was 0.1%. All transgenic combinations were entrained at 25°C under LD. Thereafter, the eye imaginal discs of third instar larvae of the genotype, w;GMR-GAL4/UAS-xbp1-EGFP;UAS-hGBA/TM6B were analyzed immunohistochemically, heads from three-day-old males with the w;GMR-GAL4/CyO;UAS-hGBA genotype were analyzed by quantitative RT-PCR and three-day-old males (Genotype: w;GMR-GAL4/CyO;UAS-hGBA) were analyzed using scanning electron microscopy.

### Statistical analysis

We verified differences in variance of the sizes of ocelli using dispersion analysis (Levene's test). Other Statistical findings were analyzed using Student's t test. The statistical significance of a difference between each transgenic combination was determined on the basis of a P-value <0.05. P-values of <0.05, 0.01 or 0.001 are described as *P<0.05, **P<0.01, or ***P<0.001, respectively.

## Results

### Generation of transgenic flies carrying hGBA variants

**Figure 1:**
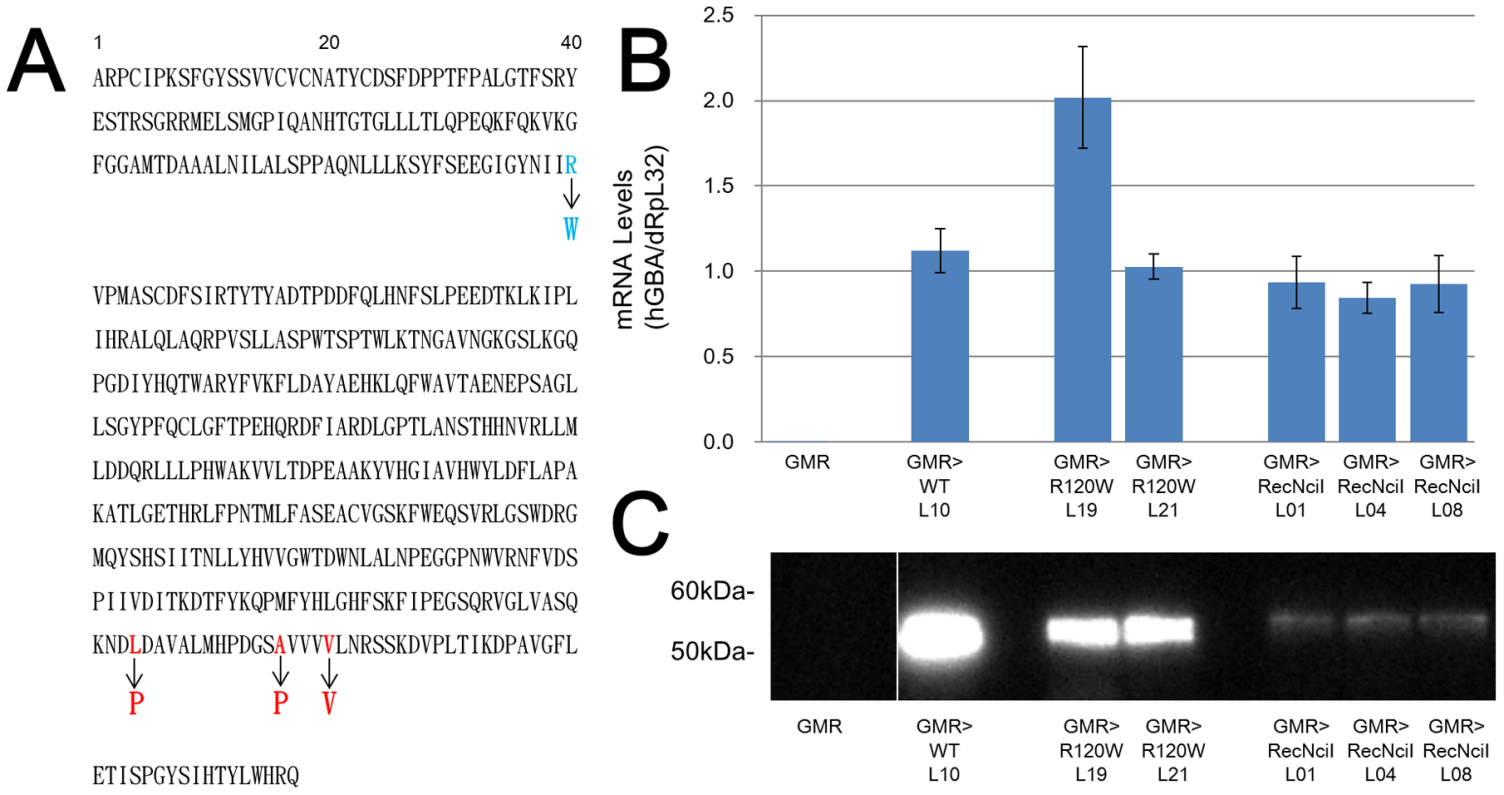
Generation of transgenic flies carrying hGBA variants. (A) Sequence of hGBA. Blue and red fonts show R120W and RecNciI mutations, respectively. (B) Expression levels of hGBA mRNA confirmed by quantitative RT-PCR (n  =  about 30 fly heads per transgenic combination) with dRpL32 as internal control. Error bars represent SE. (C) Levels of hGBA protein confirmed by Western blotting (n  =  about 100 fly heads per transgenic combination). Total amounts of hGBA protein were decreased in hGBA^R120W^, and significantly decreased in hGBA^RecNciI^ transgenic combinations, compared with hGBA^WT^ transgenic combination.

We introduced wild type hGBAs (hGBA^WT^) as well as hGBAs with R120W (hGBA^R120W^) and RecNciI (hGBA^RecNciI^) mutations into *Drosophila* to investigate molecular mechanism of GD. Figure 1A shows the amino acid sequences of the normal and mutated hGBAs seen in patients. The R120W mutation exerts mild effects [3], whereas RecNciI is associated with acute neurological abnormalities [7], [9]. We ligated the UAS promoter to hGBA to use the GAL4-UAS system that allows targeted, tissue-specific gene expression when transgenic flies bearing a UAS transgene are crossed with fly lines that express GAL4 [28]. One hGBA^WT^ (hGBA^WT L10^ where 10 is the line number), two hGBA^R120W^ (hGBA^R120W L19^, hGBA^R120W L21^) and three hGBA^RecNciI^ (hGBA^RecNciI L01^, hGBA^RecNciI L04^, hGBA^RecNciI L08^) lines of flies were generated.

We crossed each line with the GMR-GAL4 line, which drives the gene downstream of UAS in all *Drosophila* eye cells posterior to the furrow, including photoreceptor neurons and pigment cells [29]. The findings of quantitative RT-PCR and Western blotting showed that the transgenic flies expressed various levels of mRNA and proteins (Figure 1B and C). Protein expression was almost identical between the two hGBA^R120W^ and the three hGBA^RecNciI^ transgenic combinations. Western blotting showed a significant decrease in the total amount of hGBA protein in the hGBA^RecNciI^ transgenic combinations compared with the other transgenic combinations, because the RecNciI mutation includes L444P that is associated with protein degradation in patients with GD [30].

### Expression of hGBA carrying the RecNciI mutation causes neurodevelopmental defects in the *Drosophila* eye

**Figure 2:**
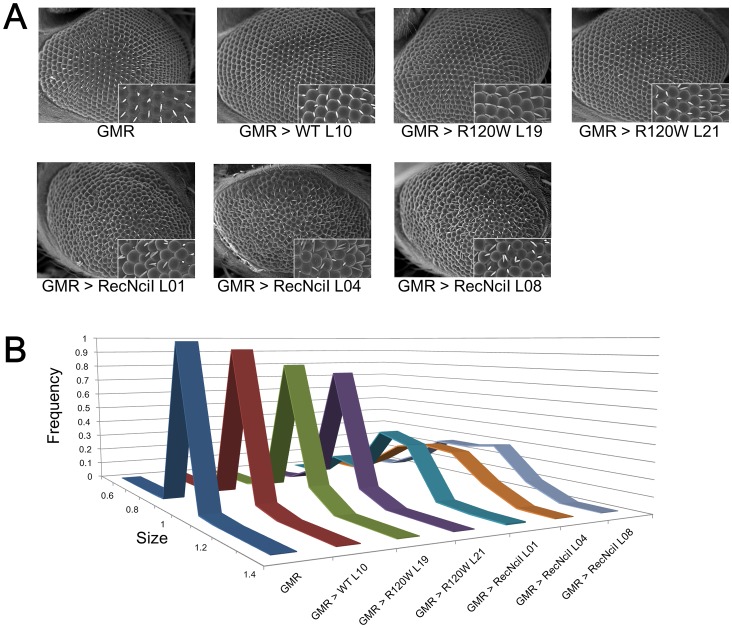
Neurodevelopmental defects in the *Drosophila* eye caused by expression of hGBA carrying the RecNciI mutation. We investigated the effects of overexpression to mutated hGBAs in fly eyes. (A) Phenotype of eyes overexpressing hGBA^WT^ transgenic combination do not significantly differ from those of GMR control. Phenotype of eyes overexpressing hGBA^R120W^ transgenic combinations occasionally differed in terms of morphology in some flies compared with control. Eye morphology is obviously affected in hGBA^RecNciI^ transgenic combinations compared with control. (B) Size histograms of ocelli in transgenic combinations (n = 3–5 flies each, about 100 ocelli each). Dispersion analysis showed obvious differences in variance of the sizes of ocelli between the hGBA^RecNciI^ transgenic combinations and the GMR control (F = 29.50–37.19; P<0.001; Levene's test).

We investigated morphological phenotypes using scanning electron microscopy to examine ectopic expression of mutated hGBAs in *Drosophila* eyes (Figure 2A). This is useful for observing the effects of expressed genes that are associated with neurodegenerative disease [17]–[21]. Overexpressing the hGBA^WT^ gene and hGBA^R120W^ gene in the eyes of the *Drosophila* transgenic combinations slightly affected eye morphology. In contrast, all hGBA^RecNciI^ transgenic combinations had an extreme, rough-eye phenotype. Dispersion analysis revealed obvious differences in variance of the sizes of ocelli between the hGBA^RecNciI^ transgenic combinations and the GMR control (Figure 2B). These results showed that hGBA with the RecNciI mutation was observed the most severe phenotype of the neurodevelopmental defects.

### Endoplasmic reticulum (ER) stress is detected in hGBR transgenic flies

We investigated whether or not the hGBA expressing transgenic flies show ER stress by using the ER stress marker, xbp1-EGFP, in which EGFP is expressed in frame only after ER stress [31].

**Figure 3:**
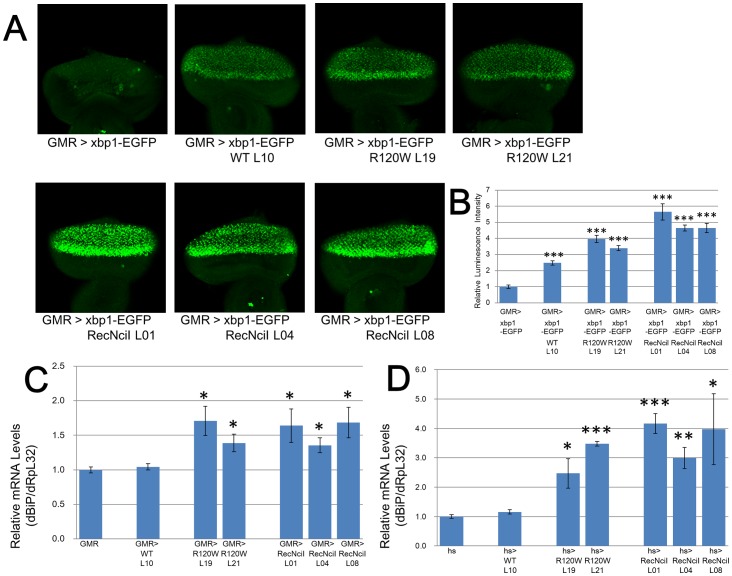
Endoplasmic reticulum (ER) stress detected in the mutated hGBA induced *Drosophila* eye. We used xbp1-EGFP as an ER stress marker in which EGFP is expressed in frame only after ER stress [31]. (A) Weak fluorescence is generated in eye imaginal discs expressing the hGBA^WT^ construct. The eye imaginal discs of hGBA^R120W^ transgenic combinations emitted more fluorescence than that of hGBA^WT^ transgenic combination. The eye imaginal discs of hGBA^RecNciI^ transgenic combinations emitted the most intense fluorescence. (B) Values generated by different transgenic combinations with fixed quantities of fluorescence intensity (n = 7–15 eye imaginal discs from third instar larvae per transgenic combination). Error bars represent SE. *Significant difference compared with values from GMR control (***P<0.001; Student's t test). (C) Endoplasmic reticulum stress marker gene, dBiP (major ER chaperone) mRNA expression in hGBA^R120W^ and hGBA^RecNciI^ transgenic combinations was upregulated (n  =  about 30 fly heads per transgenic combination). Internal control was dRpL32. Error bars represent SE. *Significant difference compared with GMR control (*P<0.05; Student's t test). (D) High levels of hGBAs are expressed in whole bodies of heat-shocked flies. Expression levels of dBiP mRNA of hGBA^R120W^ and hGBA^RecNciI^ transgenic combinations were also upregulated (n  =  about 30 flies per transgenic combination). Internal control was dRpL32. Error bars represent SE. *Significant difference compared with hs control (*P<0.05; **P<0.01; ***P<0.001; Student's t test).

We produced experimental fly combinations containing GMR-GAL4, UAS-hGBA and UAS-xbp1-EGFP and then evaluated the levels of EGFP fluorescence in the eye imaginal discs of third larval instar (Figure 3A). The hGBA^RecNciI^ transgenic combinations showed high fluorescence intensity. Fluorescence intencity was detected in the order of hGBA^RecNciI^ > hGBA^R120W^ > hGBA^WT^ expressing flies. Figure 3B summarizes fluorescence intensity. These results correlated well with the levels of morphological defects in the eyes of transgenic flies, suggesting that ER stress is one of main factors of the morphological abnormalities detected in hGBR transgenic flies.

To confirm the above findings, we evaluated the expression of another ER stress marker, dBiP gene, which is a major ER chaperone [32]. Quantitative RT-PCR showed that dBiP mRNA expression in the hGBA^R120W^ and hGBA^RecNciI^ transgenic combinations was upregulated 1.3–1.7-fold (Figure 3C). We confirmed these findings using a different driver, and crossed flies with the hs-GAL4 driver with UAS-hGBA flies that express high levels of dBiP mRNA throughout the body when heat-shocked. Expression levels of dBiP mRNA were 2.5–4.2-fold higher in the hGBA^R120W^ and hGBA^RecNciI^ transgenic combinations than in the control and hGBA^WT^ transgenic combinations (Figure 3D). These data suggest that mutated hGBAs cause ER stress not only in the eyes, but also in the whole body of *Drosophila*.

### Ambroxol can recover the morphological defects and decrease ER stress in hGBA transgenic flies

**Figure 4:**
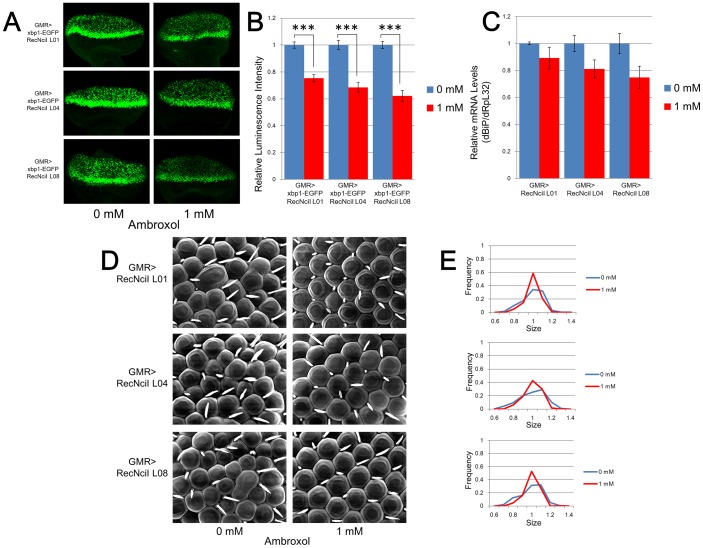
Feeding of ambroxol ameliorates neurodevelopmental defects and ER stress in the mutated hGBA induced *Drosophila* eye. Ambroxol can recover morphological defects and decrease ER stress in transgenic flies. (A) Less fluorescence emitted by the eye imaginal discs of hGBA^RecNciI^ transgenic combinations treated with, than without 1 mM Ambroxol. (B) Values generated by different transgenic combinations at fixed quantities of fluorescence intensity (n = 12–43 eye imaginal discs of third instar larvae per transgenic combination). Error bars represent SE. *Significant difference compared with controls (all without Ambroxol) (***P<0.001; Student's t test). (C) Ambroxol (1 mM) decreases expression levels of dBiP mRNA in the heads of hGBA^RecNciI^ transgenic combinations (n  =  about 30 fly heads per transgenic combination). Internal control was dRpL32. Error bars represent SE. (D) Eye phenotypes of hGBA^RecNciI^ transgenic combinations incubated without or with 1 mM Ambroxol. Size and shape of ocelli were uniform, and layout uniformity was more similar to that of normal fly eyes treated with 1 mM Ambroxol. (E) Size histograms of ocelli in hGBA^RecNciI^ transgenic combinations treated with or without 1 mM Ambroxol. (n = 6–10 flies per transgenic combination; about 400 ocelli each). Dispersion analysis showed significant differences from hGBA^RecNciI^ transgenic combinations treated with and without 1 mM Ambroxol (F = 2.07–3.35; P<0.001; Levene's test).

Ambroxol is an FDA-approved expectorant that enhances the stabilization and trafficking of mutated GlcCerase and it works as a pharmacological chaperone in fibroblasts from patients with GD [25], [30]. We therefore tested that Ambroxol can decrease ER stress in hGBA transgenic flies with standard fly food containing Ambroxol. We evaluated EGFP fluorescence intensity in the eye imaginal discs of third instar larvae and dBiP mRNA expression in three-day-old adult male heads. Ambroxol decreased EGFP fluorescence intensity (Figure 4A and B) and dBiP mRNA expression in hGBA^RecNciI^ transgenic combinations (Figure 4C). These data indicated that Ambroxol can decrease ER stress in *Drosophila* with the RecNciI mutation. We also investigated whether or not Ambroxol affects the morphological defects in hGBA^RecNciI^ transgenic combinations. The size, shape and layout of ocelli in hGBA^RecNciI^ transgenic combinations fed with Ambroxol were more uniform (Figure 4D and E), indicating that Ambroxol can recover morphological defects. These results suggest that decreasing ER stress can alleviate the morphological defects in hGBA^RecNciI^ transgenic combinations.

## Discussion

### Neurodevelopmental defects in *Drosophila* eyes caused by hGBA with RecNciI mutation

Here, we showed that hGBA with the RecNciI mutation, which caused type 2 GD (acute neurological abnormalities in humans), showed severe neurodevelopmental defects in *Drosophila* eyes. The primary defect in GD is an obvious deficiency in the activity of the lysosomal enzyme GlcCerase [33]. Deficiencies in GlcCerase result in the accumulation of its lipid substrate GlcCer in the lysosomal compartment of macrophages [10]. The defects associated with GD are thought to be caused by GlcCer accumulation. In fact, mouse models of GD based the study on the notion that GD phenotypes are caused by accumulated stored GlcCer. Therefore, mutations or deletions were constructed from the endogenous homologous genes of mouse genome. In some cases, GlcCerase variants are retained to various degrees in the ER as seen in cells of patients with GD [16]. These findings suggested that mutated GlcCerase itself is toxic, but this is yet to be confirmed at molecular level. Our *Drosophila* transgenic lines can serve as a powerful tool for investigating molecular mechanisms of neurodegeneration as well as novel therapeutic targets of GD, because our work suggests that ER stress, due to misfolding of the GlcCer protein, may be a contributory factor in the pathology of GD.

### Endoplasmic reticulum (ER) stress is a key mechanism of neurodevelopmental defects

We found here that mutated hGBAs cause ER stress as well as neurodevelopmental defects in *Drosophila* eyes, which suggest that protein products of GlcCerase might be toxic to the ER. This findings suggest that mutated GlcCerase could serve as a new therapeutic target for type 2 GD. ER stress contributes to neurodegeneration across a range of neurodegenerative disorders [24] and it might also be responsible for neurodegeneration in the eyes of *Drosophila* transfected with hGBAs, especially when they harbor the RecNciI mutation that is associated with acute neurological abnormalities in GD patients [7], [9]. Previous reports indicated that ER stress is a common mediator of apoptosis in both neurodegenerative and non-neurodegenerative lysosomal storage disorders including GD [34]. Unfolded protein response activation observed in fibroblast cells from neuronopathic GD patients might be a common mediator of apoptosis in neurodegenerative lysosomal storage disorders. This suggests that mutated hGBAs may cause apoptosis through ER stress in *Drosophila* eyes.

### Ambroxol ameliorates neurodevelopmental defects and decreases ER stress induced by mutant hGBA expression in *Drosophila* eye

Ambroxol is known as a pharmacological chaperone for mutant glucocerebrosidase including the L444P point mutation [30]. Our results showed that Ambroxol can decrease ER stress and ameliorate neurodevelopmental defects in *Drosophila* with the RecNciI mutation. The complex allele RecNciI also includes L444P point mutation. The data suggests that Ambroxol acts as a pharmacological chaperone for the RecNciI GlcCerase variant in *Drosophila* eye. As ER stress contributes to neurodegeneration across a range of neurodegenerative disorders [24], Ambroxol may have an important use in ameliorating neurodegeneration in GD patients.
