# Correction: Expression of Human Gaucher Disease Gene GBA Generates Neurodevelopmental Defects and ER Stress in *Drosophila* Eye

**DOI:** 10.1371/journal.pone.0135619

**Published:** 2015-08-19

**Authors:** Takahiro Suzuki, Masami Shimoda, Kumpei Ito, Shuji Hanai, Hidenobu Aizawa, Tomoki Kato, Kazunori Kawasaki, Terumi Yamaguchi, Hyung Don Ryoo, Naoko Goto-Inoue, Mitsutoshi Setou, Shoji Tsuji, Norio Ishida

In the Materials and Methods section, under the subheading Western blotting, the second sentence incorrectly refers to 0.5M EDTA. The correct concentration is 10mM EDTA. The correct sentence is: All transgenic combinations were entrained at 25°C under LD, and then the heads of flies with the w;GMR-GAL4/CyO;UAS-hGBA genotype collected at 11.00 a.m. were homogenized in extraction buffer containing 20 mM HEPES (pH 7.5), 100 mM KCl, 5% glycerol, 100 mM Na3VO4, 10 mM EDTA, 0.1% Triton-X, 10 mg/mL antipain, 10 mg/mL pepstatin-A, 10 mg/mL leupeptin, 24 TIU/mL aprotinin and 0.1 M phenylmethyl-sulfonyl-fluoride (PMSF).
